# Aging, Social Determinants of Health in the Case of Persons With Disabilities and Refugees

**DOI:** 10.1093/geroni/igab046.306

**Published:** 2021-12-17

**Authors:** Jean-Francois Trani

**Affiliations:** Brown school, Washington University in St Louis, Missouri, United States

## Abstract

Structural and social determinants of health differentially impact on social groups. Among those particularly disadvantaged during the life course are both persons with disabilities and refugees. Because of the way society treats these two populations, both persons with disabilities and refugees may face physical, social, economic and environmental barriers that impede them from benefiting from the same opportunities accessible to other social groups. As a result, these populations have less access to education, higher unemployment, are more likely to be deprived and excluded from social benefits. In other words, public stigma —prejudice and discrimination voiced and practiced by the general population— translates to a life course characterized by daily stressors that result in a higher likelihood of cognitive disorders and dementia. Measuring and analyzing SSDoH inclusive of disability and refugee experiences are essential to efforts aimed at recruitment and retention and knowledge generation in ADRD research.

